# A new approach to quantification of mAb aggregates using peptide affinity probes

**DOI:** 10.1038/srep42497

**Published:** 2017-02-10

**Authors:** Crystal S. F. Cheung, Kyle W. Anderson, Pooja M. Patel, Keale L. Cade, Karen W. Phinney, Illarion V. Turko

**Affiliations:** 1 Biomolecular Measurement Division, National Institute of Standards and Technology, Gaithersburg, MD 20899, USA; 2 Institute for Bioscience and Biotechnology Research, Rockville, MD 20850, USA

## Abstract

Using mAbs as therapeutic molecules is complicated by the propensity of mAbs to aggregate at elevated concentrations, which can lead to a variety of adverse events in treatment. Here, we describe a proof-of-concept for new methodology to detect and quantify mAb aggregation. Assay development included using an aggregated mAb as bait for screening of phage display peptide library and identifying those peptides with random sequence which can recognize mAb aggregates. Once identified, the selected peptides can be used for developing quantitative methods to assess mAb aggregation. Results indicate that a peptide binding method coupled with mass spectrometric detection of bound peptide can quantify mAb aggregation and potentially be useful for monitoring aggregation propensity of therapeutic protein candidates.

Molecular interactions that occur in high concentration formulations of monoclonal antibodies (mAbs) increase the propensity of antibody molecules to undergo aggregation. Aggregation is particularly concerning because of the suspected immunogenicity of antibody aggregates^1^,^2^. In addition, differences in biological activity of the aggregated mAbs compared to the activity of the monomeric mAbs can significantly impair the potency of a therapeutic^3^,^4^. mAb aggregates arise in a variety of forms including reversible non-covalent, irreversible non-covalent, and irreversible covalent species^4^,^5^,^6^,^7^,^8^. Sizes of these aggregates broadly vary from a few nanometers to hundreds of microns and this presumably affects biological properties of mAbs. Little is known about the morphology of these aggregates, which likely ranges from irregular or spherical particulates to ordered fibrils. A variety of analytical techniques have been used to assess the size and number of aggregates in samples of purified mAbs^8^,^9^,^10^,^11^,^12^,^13^,^14^. Unfortunately, most of these techniques are semi-quantitative in their nature and cannot provide absolute quantitative data. Furthermore, analytical techniques to quantify aggregates with different morphologies in complex mixed samples do not currently exist. Ideally, an analytical method that could discern between different aggregate morphologies and provide quantitation of these aggregate morphologies in complex samples would be highly beneficial in both research and clinical settings.

In the present work, we reasoned that protein-protein interfaces formed by mAb aggregation could be selectively recognized by short peptides with random amino acid sequence. Protein-protein interfaces that do not exist in monomeric mAbs, but are present in aggregated mAbs, can be a target for high affinity peptide probes. This idea directed our attention to peptide phage display technology, which is a powerful tool in the identification of ligands with novel functions^15^,^16^,^17^. Peptides bound to aggregate interfaces can be selected from a complex mixture of billions of displayed peptides on the phage and further enriched through the biopanning process. Once identified, the selected peptides can be used for developing quantitative methods to assess mAb aggregation.

## Results

To quantify mAb aggregation, we developed a new binding method with mass spectrometry detection. The binding method relies on a short peptide which can recognize mAb aggregates versus mAb monomers. The process of identifying candidate peptides included several steps, which are described in the next section.

**Figure 1:**
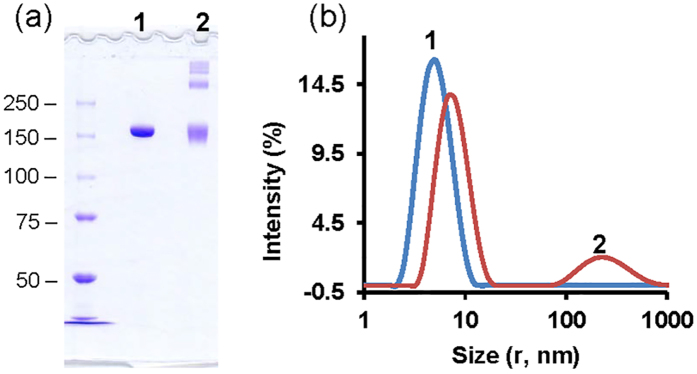
SDS-PAGE (**a**) and DLS (**b**) for aggregates of NISTmAb cross-linked with BS^3^. Molecular mass standards for SDS-PAGE are shown on the left in kDa. Data are presented for two samples: control (1) and cross-linked (2).

In the first step, a prototype mAb, NIST RM8670 (NISTmAb)^18^, was agitated for 3 days at room temperature. The generated aggregates were then cross-linked with an amine-reactive cross-linker, bis(sulfosuccinimidyl)suberate (BS^3^). Cross-linking was used to prevent dissociation of aggregates during the following phage display panning. It is important to note that to avoid forced aggregation of NISTmAb during cross-linking, a soft single treatment with BS^3^ was used. This supposes stapling existing aggregates rather than forcing formation of new aggregates. Figure 1a shows a non-reduced SDS-PAGE of control and cross-linked NISTmAb. The cross-linked species are clearly observed and represented by a number of aggregated material populations. The cross-linked bands are discrete and there is no protein smear, supporting non-excessive treatment with BS^3^. Dynamic light scattering (DLS) analysis of these samples is shown in Fig. 1b. Untreated NISTmAb contains one major population by intensity with hydrodynamic radius 5.2 nm. Cross-linked NISTmAb contains two major populations by intensity with hydrodynamic radius 6.8 nm and 230 nm, respectively. Increase in hydrodynamic radius of NISTmAb monomer after cross-linking may point to the presence of intra-molecular cross-links and, in general, the cross-linked NISTmAb shows high polydispersity suggesting the presence of multiple aggregated populations.

**Figure 2:**
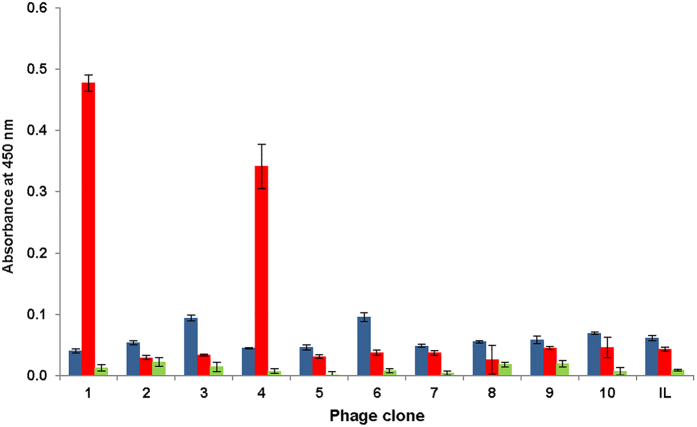
ELISA for selected phage clones. The binding affinities toward control NISTmAb (blue bars), NISTmAb aggregates generated by agitation (red bars), and bovine serum albumin (green bars) are shown. IL stands for insertless phage clone. The experiments were performed with three analytical replicates and data are presented as the mean ± SD.

In the second step, a sample of the cross-linked NISTmAb was used as bait to screen a Ph.D.−12 phage display peptide library. The bait represents a mixture of monomer and cross-linked aggregated NISTmAb; this was also the only occasion when cross-linked sample was used. Phage display panning was performed in a subtractive manner. The library was initially depleted with control non-aggregated NISTmAb using three iterative cycles. The unbound phage was then screened on cross-linked NISTmAb aggregates, again using three iterative cycles of phage binding, elution, and amplification. After these rounds of panning, the single phage plaques were randomly picked up and amplified, and phage DNA was submitted for sequencing. In parallel, the ability to bind NISTmAb aggregates was evaluated using phage enzyme-linked immunosorbent assay (ELISA) for all selected phage clones. Figure 2 shows summarized data of binding ability of the 10 selected phage clones toward albumin (green bars), control non-aggregated NISTmAb (blue bars), and aggregated by agitation NISTmAb (red bars). An insertless phage, which does not have the peptide DNA sequence inserted in its genome and therefore does not display peptide sequence on its surface, is also shown and marked as IL. Clones #1 and #4 show respectively 9-fold and 7-fold better binding toward aggregated NISTmAb, compared to their binding toward control non-aggregated NISTmAb. In fact, these two clones show the lowest (compared to other clones) affinity toward non-aggregated NISTmAb. The result here suggested that phage clones #1 (displaying peptide RDYHPRDHTATWGGG, P02-C01) and #4 (displaying peptide GNNPLHVHHDKRGGG, P02-C04) possess preferential binding to NISTmAb aggregates. We also would like to mention that in Ph.D.−12 phage display peptide library, a four amino acid residue sequence (GGGS) connects each displayed peptide and pIII protein of phage. We did not know whether these amino acid residues may add to binding affinity of specific clones and decided to include GGG residues in the final peptide sequences shown in Table 1.

Comparison of P02-C01 and P02-C04 using computational tools, such as ProtParam (http://web.expasy.org/protparam/), BLAST (http://blast.ncbi.nlm.nih.gov/Blast.cgi), and LALIGN (http://www.ch.embnet.org/software/LALIGN_form.html) did not reveal common features, except that both peptides are rather hydrophilic with grand average of hydropathicity being −1.73 and −1.58, respectively. This prompted us to select the one with highest affinity, P02-C01 for further development of binding assay.

**Figure 3:**
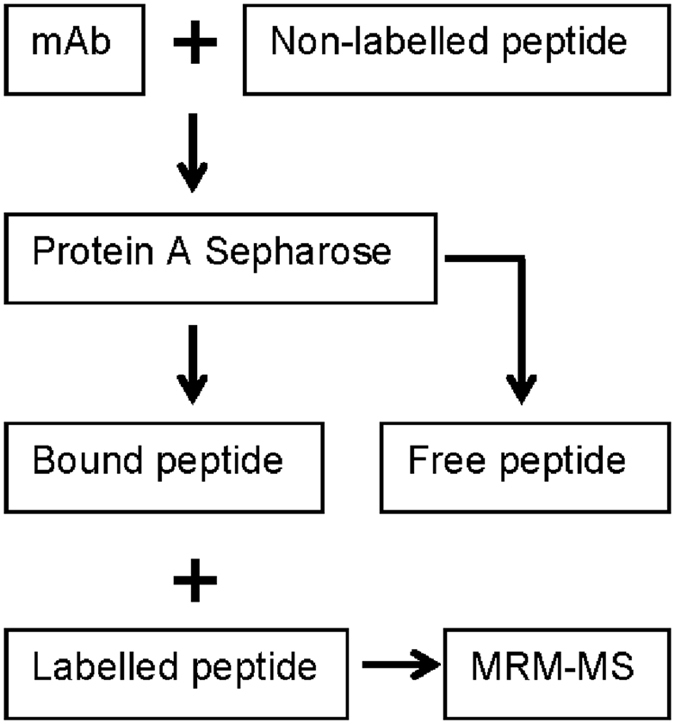
Scheme of the peptide binding assay.

In the third step, we used a traditional idea of a binding assay, which includes incubation of protein with ligand, then separation of protein-bound and free ligands, and finally quantification of protein-bound ligand. The scheme of our specific binding assay is depicted in Fig. 3. For this pilot study, we ordered chemical synthesis of non-labeled P02-C01 (RDYHPRDHTATWGGG) and stable isotope-labeled P02-C01 (RDYH*PRDHTATWGGG) carrying *P, [^13^C_5_, ^15^N_1_]-Pro residue. Control and aggregated under various conditions NISTmAbs were allowed to interact with a 100-fold molar excess of non-labeled peptide P02-C01. Following 30 min incubation on ice, the bound and free P02-C01 were separated by passing the mixture through the spin column with protein A Sepharose, which captured the complexes of P02-C01 with NISTmAb. To minimize loss of bound peptide (if any) during the column separation, the spinning was performed at +4 °C for a limited time of 15 sec. Samples eluted from protein A Sepharose were supplemented with a known amount of labeled P02-C01 and analyzed using multiple reaction monitoring (MRM) assay. The MRM assay relies on liquid chromatography coupled with triple quadrupole mass spectrometry. The high specificity of the MRM assay is achieved by monitoring multiple transitions (precursor ion – product ion pairs) for each target peptide. Quantification is performed by comparing the chromatographic peak area of a transition from an unlabeled native peptide to the corresponding transition from a stable isotope-labeled internal standard^19^,^20^.

By replacing protein A Sepharose spin column with a strong cation exchange spin column, this binding assay can also be adapted to virtually any protein. Thermally aggregated lysozyme is broadly used as a model protein in structural studies of pathway of aggregate formation and conditions of lysozyme aggregation are well described^21^. In our work, we utilized lysozyme in control experiments to investigatenon-specific binding of P02-C01 to proteins other than immunoglobulin G (IgG).

**Figure 4:**
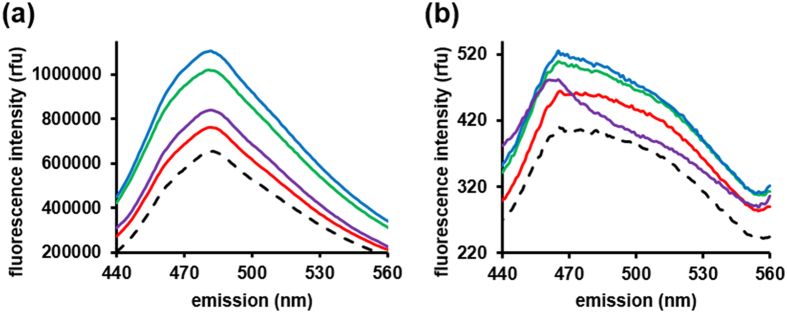
Fluorescence emission spectra of 40 μmol/L ThT (**a**) or 40 μmol/L ANS (**b**) in the presence of 10 μmol/L NISTmAb or 10 μmol/L IgG1-κ from human plasma. Black dash line shows control NISTmAb. Colored lines represent various aggregates: agitated NISTmAb (green line), NISTmAb oxidized at room temperature (red line), NISTmAb oxidized at 37 °C (blue line), and IgG1-κ from human plasma oxidized at room temperature (purple line).

The actual measurements were performed for three groups of samples. The first group included control and aggregated NISTmAb. Aggregation was achieved by agitation for 3 days at room temperature and by oxidation at room temperature or 37 °C. The second group was designed to address the possibility that P02-C01 might recognize aggregation of IgGs other than NISTmAb. This sample group included control and aggregated samples (obtained by oxidation at room temperature) derived from a human IgG1-κ (Sigma-Aldrich). The third group was designed to verify whether P02-C01 would recognize aggregation of a protein other than IgG. This last group includes control and thermally aggregated lysozyme. Although the treatments described above are traditionally used to cause and study aggregation of mAbs^22^,^23^, actual aggregation of NISTmAb was validated with two fluorescence assays, based on thioflavin T (ThT) and 8-anilino-1-naphthalenesulfonate (ANS) binding (Fig. 4). Both fluorescent dyes are broadly used to detect mAb aggregation^24^,^25^,^26^. ThT recognizes amyloid-type (β-sheets) protein aggregates. Aggregate formation is detected by the increase in the fluorescence intensity at around 482 nm. The native monomer mAb structure is mainly β-sheet and ThT binding to control NISTmAb is well observed (Fig. 4a). However, all conditions used to aggregate NISTmAb shows higher fluorescence which is consistent with favorable ThT binding to aggregates (Fig. 4a). ANS is typically used to study protein conformational changes. Since conformational changes are an intrinsic feature of mAb aggregation, ANS was found particularly suitable for detection of mAb aggregation^24^,^25^,^26^. Excitation of the free ANS at 350–380 nm results in a low fluorescence with a maximum at 545 nm while binding of ANS to the hydrophobic regions of a protein causes an increase in fluorescence intensity. The emission spectrum of the ANS-protein complex is also shifted to a broad maximum at around 470 nm. As expected, the binding of ANS to control NISTmAb (Fig. 4b) shows an emission peak already shifted to around 470 nm. However, all conditions used to aggregate NISTmAb again shows higher fluorescence, pointing to the presence of aggregated forms of protein (Fig. 4b).

To perform the MRM assay, the optimum MRM transitions for P02-C01 were determined by generating a table of the observed +2 charge precursor ions with the corresponding +1 charge *b* and *y* fragment ions, and the +3 charge precursor ions with the corresponding +1 and +2 charge *b* and *y* fragment ions. Only ions with *m/z* values greater than the precursor ion *m/z* were included in the table, because these fragments tend to be more intense and there is less noise in this region of the spectrum. The LC-MS/MS was set to select all the MRM transitions in the table. The intensities of the MRM transitions were recorded and the six most intense transitions were used for further quantification (Table 2).

**Figure 5:**
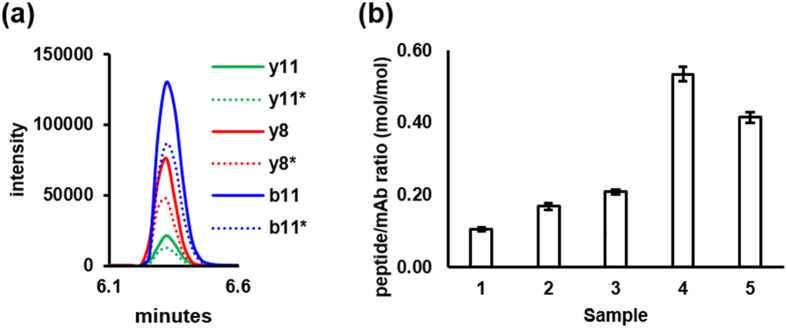
Quantification of aggregation in P02-C01 binding MRM assay. (**a**) Extracted ion chromatograms of representative transitions monitored for P02-C01. Non-labeled (solid line) and labeled (dotted line) transitions are color coordinated. Data are presented for NISTmAb oxidized at 37 °C (sample 4). (**b**) Control NISTmAb (sample 1), agitated NISTmAb (sample 2), NISTmAb oxidized at room temperature (sample 3), NISTmAb oxidized at 37 °C (sample 4), and IgG1-κ from human plasma oxidized at room temperature (sample 5) are shown. The data are normalized to the molar amount of total IgG in the assay and presented as a mol/mol ratio of bound P02-C01 to IgG. The experiments were performed in duplicate with three analytical replicate injections and data is presented as the mean ± SD.

Figure 5 summarizes quantification of aggregation under various conditions. The data for lysozyme are not shown because there was no P02-C01 binding to either control or aggregated lysozyme. Figure 5a shows typical extracted ion chromatograms for three representative transitions (y^8^, y^11^, and b^11^) monitored for P02-C01 and demonstrates that individual non-labeled/labeled ratios measured for P02-C01 are highly similar in MRM assay. Knowing the concentration of labeled P02-C01, these ratios were converted in concentration of unknown non-labeled P02-C01. In whole, the data for NISTmAb and IgG1-κ are normalized to the molar amount of total IgG in the assay and presented as a mol/mol ratio of bound P02-C01 to IgG (Fig. 5b). There is a background binding of this peptide to the untreated control (sample 1), which presumably does not have aggregates or their level should be negligibly low. However, all treated and aggregated samples (samples 2–5) have higher than control P02-C01 binding. This data is indicative that, in addition to high affinity of P02-C01 to treated aggregated NISTmAb, P02-C01 also has a low affinity to control non-aggregated NISTmAb.

**Figure 6:**
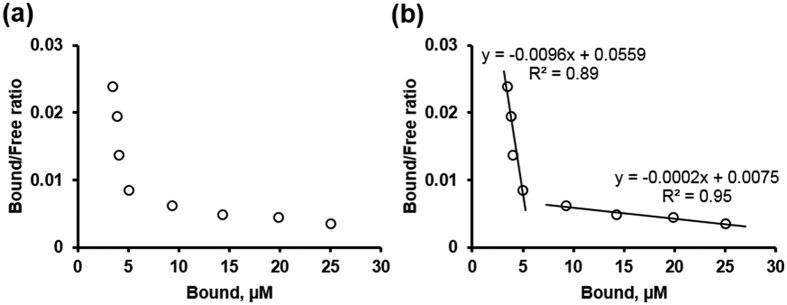
Scatchard plot for P02-C01 binding to NISTmAb stressed by agitation. (**a**) Distribution of experimental data points. (**b**) Fitting of experimental data points into two binding sites with different K_d_ values.

Figure 6 shows the Scatchard plot, which displays specific binding of P02-C01 to the NISTmAb stressed by agitation versus the ratio of specific binding to the concentration of free P02-C01 used in saturation binding experiment. The shape of the Scatchard plot must be linear for a system which has one identical and independent set of binding sites. The shape of the Scatchard plot for P02-C01 binding does not meet this requirement (Fig. 6a), however the shape does fit well for a system with two sets of binding sites^27^. In Fig. 6b, the data were divided into two pools and two straight lines were plotted. The equilibrium binding constants (K_d_) were estimated as −1/slope. This analysis pointed to existence of two binding sites for P02-C01 with apparent K_d_ values 104 μM and 5 mM. Although this is not the most accurate approach to measure K_d_, the Scatchard analysis succeeds in clearly presenting two distinct binding sites with suitable calculation of respective K_d_ values. Furthermore, the two-site binding results concur well with the observed binding affinity of P02-C01 to both control non-aggregated and aggregated NISTmAb (Fig. 5).

## Discussion

The development of mAb therapeutics is currently the fastest growing segment of the biopharmaceutical industry and mAb aggregation is a commonly encountered problem in all levels of development, including the manufacturing process, product efficacy/delivery and also patient safety. The mAb aggregation is driven by a number of factors which can result in different forms of aggregates. Most of the currently available aggregation measurement methods are not well suited to the quantitative analysis of complex protein samples, such as aggregated mAbs, and there is a significant need in the industry for methods based on new principals to recognize/quantify different aggregate species. Overall, the main aim of this study was to validate the hypothesis that peptide with random sequence can act as an affinity reagent to recognize mAb aggregation and can be used to quantify mAb aggregates.

The first step was based on the screening of a commercially available phage display library of 12-amino-acid-long peptides using aggregated NISTmAb as the bait. The aggregated NISTmAb was also cross-linked to prevent dissociation of aggregates during the process of library screening. Screening resulted in two prominent hits, which possess obviously selective binding to aggregated NISTmAb in comparison to control NISTmAb in an ELISA assay (Fig. 2). Binding to albumin in this assay was used to assess the level of total non-specific binding. Finally, we selected one hit, P02-C01 peptide RDYHPRDHTATWGGG (Table 1), to be used for developing the quantitative assay.

The quantitative assay is based on the general idea that after incubation of ligand and protein, the protein-bound and free ligands can be separated and the protein-bound ligand quantified. There are many ways this broad idea can be implemented, but the two critical components are: 1) a method for separation of bound and free ligands, and 2) a method of detection of the protein-bound species. Since our goal was to quantify peptide bound to IgG, commercial spin columns with protein A Sepharose were a straightforward choice for separation of IgG-bound peptide and free peptide. More universal protein binding resins, such as cation exchange resins, will work as well. This was demonstrated in control experiments with aggregated lysozyme using strong cation exchange spin columns from Thermo Scientific to separate bound and free species. The purpose of using aggregated lysozyme was to verify ability of P02-C01 to specifically recognize aggregation of IgG rather than aggregation of any protein. The binding assay based on cation exchange columns also worked well for NISTmAb (data not included). At the same time, there was no detectable binding of P02-C01 to control or aggregated lysozyme, confirming that P02-C01 binding is IgG-specific. Additional evidence for IgG specificity came from measurements performed with human IgG1-κ purchased from Sigma-Aldrich (Fig. 5). Human IgG1-κ was intentionally selected for comparison since NISTmAb is also IgG1-κ. MRM measurements validate the fact that P02-C01 recognizes not just NISTmAb aggregation, but aggregation of IgG (or at least IgG1-κ) as a protein class. While P02-C01 is not likely binding all aggregate morphologies in IgG, it does appear to bind at least one aggregate morphology that is conserved in similarly treated NISTmAb and IgG1-κ aggregate samples. Expansion of this work could include different peptide probes, each recognizing a specific morphology conserved in IgG-κ aggregates, which could be multiplexed for more complete characterization of a complex sample of mixed aggregates. While we have not performed multiplexed analysis herein due to our early limitation with one peptide probe, this proof-of-concept method demonstrates the feasibility of developing a more robust multiplexed assay.

Saturation binding data in Fig. 6 also presents several points for discussion. From one side, this underlines importance of careful selection of aggregated species for phage display selection, meaning that differently aggregated and purified mAbs have to be used in individual screenings to find a panel of peptides that will cover the individual morphologies of aggregation. Our bait had both, aggregated and non-aggregated NISTmAb. As a result of this, P02-C01 has a double affinity despite depletion of library on control NISTmAb and then repeated rounds on mixed bait. From another side, it is a strong proof of overall feasibility of proposed approach. The apparent K_d_ values (104 μM and 5 mM) are very different and prove that selective binding to aggregated mAb is possible. We believe that more stringent selection can generate peptides with high affinity and selectivity to specific forms of mAb aggregates. In regards to phage display library, we envision that a library of slightly longer than 12 amino acid residues may need to be custom generated. Too long peptides can cause many experimental challenges and do not seem necessary to achieve highest affinity and selectivity, but peptides near 18 amino acid residues will presumably provide better performance.

The presented approach to analyze mAb aggregation is new and in addition to current challenges of improving affinity and selectivity of peptide probe, we also would like to discuss the future trends for this concept in general. To detect and quantify P02-C01 bound to NISTmAb, we used the MRM assay, because it is a highly selective and sensitive modern approach^18^,^19^,^28^,^29^,^30^. It was suitable to assess performance and reliability for this pilot study aiming to demonstrate the proof-of-concept. MRM assay remains a valid approach to study mAb aggregation, however it may have a limited application for high throughput analysis. Another crucial aspect that should be mentioned is that spin column separation of bound and free peptides cannot fully guarantee quantitative recovery of bound peptide. To overcome these limitations in the future, it would be beneficial if the peptide binding assay could be coupled with detection in a 96-well plate format and the assay itself will become a homogeneous assay without column separation. For example, various fluorescence-based assays, especially fluorescence polarization assays, can be successfully adapted to this purpose^31^,^32^.

In summary, we presented experimental data supporting the original hypothesis that a random sequence peptide selected from a phage display library can be used as a new affinity reagent in developing quantitative assays for mAb aggregation. Further exploration of this idea is of interest to major arenas of antibody applications.

## Method

### Preparation of protein aggregates

Aggregation of the full length NIST RM8670 (NISTmAb)^18^ was initiated by mechanical stress or chemical oxidation. For mechanical stress, 10 mg/mL NISTmAb in the 25 mmol/L His-buffer (pH 6.0) were agitated at speed 5 using Vortex-Genie 2T (Scientific Industries, Bohemia, NY) for 3 days at room temperature. Control sample of NISTmAb was kept for 3 days at room temperature without mechanical stress. For chemical oxidation, 10 mg/mL NISTmAb in the 25 mmol/L His-buffer (pH 6.0) were treated with 1 mmol/L H_2_O_2_ and 0.1 mmol/L CuCl_2_ for 1 h at room temperature or at 37 °C.

Two control proteins, IgG1-κ from human myeloma plasma (I5154, Sigma-Aldrich) and lysozyme from chicken egg white (L7651, Sigma-Aldrich), were used in this study. IgG1-κ was aggregated by chemical oxidation at room temperature at conditions used for NISTmAb. Lysozyme aggregation was induced by heating a protein solution at 60 °C for 30 min^21^.

### Chemical cross-linking and SDS-PAGE analysis

NISTmAb sample stressed by agitation was treated with 100-fold molar excess of a cross-linking reagent, bis(sulfosuccinimidyl) suberate (BS^3^) for 30 min at room temperature. The cross-linking was quenched by adding 50 mmol/L TrisHCl (pH 7.5) and incubating for 15 min at room temperature.

For non-reduced SDS-PAGE, all samples were treated with 2% SDS/6% glycerol with some bromophenol blue and then analyzed using homemade 6% SDS-PAGE gels.

### Dynamic light scattering

A Malvern ZetaSizer Nano-ZS analyser (Malvern Instruments, UK) was used for dynamic light scattering (DLS) measurements. The 4 mW HeNe laser was set at λ = 633 nm with the detector angle at 173° for backscattering measurements. The NISTmAb samples were adjusted with 25 mmol/L His-buffer (pH 6.0) to give a concentration of 1 mg/mL. His-buffer was filtered through a Millipore Millex-GV 0.22 μm PVDF filter. DLS spectra were acquired in a disposable polystyrene ultra-micro cuvette at room temperature. Each spectrum was collected over three runs consisting of twelve ten second scans. The three runs were then averaged.

### Phage display peptide library

Ph.D.−12 phage display peptide library was purchased from New England BioLabs (Ipswich, MA, USA). The library consists of M13 filamentous bacteriophage, on which five copies of a 12-amino-acid linear peptide sequence are expressed as N-terminal fusions to the minor coat protein pIII of the phage. A short linker glycine-glycine-glycine-serine (GGGS) is present between each displayed peptide and pIII protein. The M13 phage is propagated in *E. coli* host strain ER2738 (New England BioLabs) in Luria-Bertani (LB) medium containing 20 μg/mL tetracycline (Sigma-Aldrich, St. Louis, MO).

### Phage display panning

100 μg of control NISTmAb and NISTmAb stressed by agitation and cross-linked with BS^3^ were coated to a 96-well plate at 4 °C overnight. Then, 200 μL of 3% bovine serum albumin (BSA) in phosphate-buffered saline with 0.005% Tween 20 (PBST) was added to the well for non-specific blocking at room temperature for 1 h. For iterative screening, 1 × 10^11^ plaque-forming units (pfu) of naïve phage library (in 100 μL of PBST) were incubated with control NISTmAb at room temperature for 1 h with gentle shaking. Unbound phages were later removed and transferred to another well coated with control NISTmAb. This procedure was repeated once more. Following binding to the control NISTmAb three times, the unbound phage was added to the well containing cross-linked NISTmAb aggregates, and allowed to incubate at room temperature for 1 h with gentle shaking. Finally, the phage that bound poorly to the cross-linked NISTmAb aggregates was discarded by washing the wells ten times with PBST. The bound phage was then eluted with 200 μL of 0.2 mol/L glycine-HCl (pH 2.2) at room temperature for 10 minutes, and neutralized with 1/10 volume of 1 mol/L NaCl (pH 9.1). Subsequently, the eluted phage was tittered and amplified. The amplified phage pool was subjected to two additional rounds of this subtractive screening approach at a fixed phage input of 1 × 10^11^ pfu. Titters of eluted non-amplified phage after each round of selection were 1 × 10e2 pfu/μL (1^st^ round), 1.4 × 10e4 pfu/μL (2^nd^ round), and 2.1 × 10e4 pfu/μL (3^rd^ round).

### DNA sequencing

Following three rounds of panning, ten single phage plaques were randomly picked from the tittering plates. Each phage plaque was then used to infect 1 mL early-log phase *E. coli* ER2738 culture at 37 °C for 4.5 hours. The phage-containing supernatant was collected by centrifugation at 20,000 g at 4 °C for 10 minutes, followed by precipitation with 400 μL of 20% PEG/2.5 mol/L NaCl at room temperature for 20 minutes. Thereafter, the mixture was centrifuged at 20,000 g at 4 °C for 10 minutes. The phage pellet was resuspended in 400 μL of 10 mmol/L Tris-HCl (pH 8.0)/1 mol/L EDTA/4 mol/L NaI, and then incubated in 500 μL of 100% EtOH at room temperature for 20 minutes. The mixture was centrifuged at 20,000 g at 4 °C for 10 minutes to remove the supernatant. The pellet, which contains the phage DNA, was subsequently washed once with 500 μL of ice-cold 70% EtOH, centrifuged and air-dried. The purified DNA was sequenced, using the −96 gIII sequencing primer: 5′-CCC TCA TAG TTA GCG TAA CG-3′ (New England BioLabs).

### Phage ELISA

To assess the binding ability of the selected phages toward NISTmAb aggregates, 1 × 10^10^ pfu of P02-C06 phage was incubated with 50 μg of NISTmAb aggregates at room temperature for 2 hours. Wild-type M13 phage, M13KE, (New England BioLabs), which did not bear any peptide insert, was used as a negative control. After binding, the NISTmAb aggregate-coated wells were washed three times with PBST, then horseradish peroxidase-conjugated anti-M13 antibody (GE Healthcare, Piscataway, NJ) (diluted 1:5000 in 3% BSA) was added and incubated at room temperature for 1 hour. Tetramethybezidine substrate reagent (eBioscience, San Diego, CA) was subsequently added and absorbance was read at 450 nm.

### Peptide binding assay

Non-labeled P02-C01 peptide (RDYHPRDHTATWGGG) and labeled P02-C01 peptide (RDYH^*^PRDHTATWGGG) were ordered from Biomatik, Wilmington, DE, USA. ^*^P in labeled P02-C01 peptide stands for [^13^C_5_, ^15^N_1_]-Pro amino acid residue. Both peptides were 97% pure based on HPLC analysis.

Samples of 0.5 nmol of NISTmAb were mixed with 50 nmol of non-labeled P02-C01 in 40 μL of 25 mmol/L His-buffer (pH 6.0), incubated on ice for 30 min, and loaded on protein A HP SpinTrap columns (GE Healthcare) pre-equilibrated with cold 25 mmol/L His-buffer (pH 6.0). After a quick wash with 0.6 mL of cold His-buffer (150 g, 30 sec), the columns were eluted with 0.4 mL of 0.5% TFA (150 g, 30 sec). The eluates were supplemented with 200 pmol of labeled P02-C01 peptide and dried. Supplementation with labeled P02-C01 provides an internal standard for future LC-MS/MS quantification.

For saturation binding, samples of 0.5 nmol of NISTmAb stressed by agitation were mixed with increasing amounts of non-labeled P02-C01 (150, 200, 300, 600, 1500, 3000, 4500, and 6000 nmol). The binding assay was then performed as described above.

### LC-MS/MS analysis

Dried eluates were reconstituted in 30 μl of 3% acetonitrile/97% water containing 0.1% formic acid. 8 μl of sample were used for each LC-MS/MS run. Instrumental analyses were performed on a Thermo Scientific Accucore C4 column (2.1 mm × 150 mm, 2.6 μm particle) coupled to an Agilent 6490 Triple Quadrupole LC/MS system (Santa Clara, CA). Peptides were eluted over a 19-min gradient from 3% to 45% acetonitrile containing 0.1% formic acid at a flow rate of 200 μL/min. Solvent B was increased from 45% to 95% in 5 min and held at 95% for 2 min before returning to 3% to clean the column. A blank run was performed after each sample run to ensure no carryover was present before analysis of subsequent sample. Solvent A was water containing 0.1% formic acid and solvent B was 100% acetonitrile with 0.1% formic acid. The acquisition method used the following parameters in positive mode: fragmentor 380 V, electron multiplier 500 V, capillary voltage 3500 V, dwell time 120 ms, and collision energy 25 V.

### Data analysis

MRM peak area integration was performed using Skyline 3.5 (https://skyline.gs.washington.edu/labkey/wiki/home/software/Skyline/page.view?name=default). Excel was used to calculate peak area ratios. Individual peak integrations were validated manually. The peak ratios from transitions were averaged to yield the peptide ratios. All samples were prepared and analyzed in triplicate, therefore data are represented as the mean ± SD.

### Extrinsic fluorescence

For ThT experiments, 10 μmol/L NIST mAbs or IgG1-κ from human plasma were incubated with 40 μmol/L ThT in 25 mmol/L His-buffer (pH 6.0) for 3 hrs. Fluorescence measurements were performed at room temperature on FluoroMax-3 (Horiba Jobin Yvon, Edison, NJ). ThT was excited at 415 nm and its fluorescence emission was recorded between 440–600 nm. For ANS experiments, ANS was dissolved in ethanol and then freshly diluted with 25 mmol/L His-buffer (pH 6.0) to 200 μmol/L working stock solution, 10 μmol/L NISTmAbs or IgG1-κ from human plasma were then incubated with 40 μmol/L ANS in 25 mmol/L His-buffer (pH 6.0) for 30 min. Fluorescence measurements were performed at room temperature on SpectraMax M5 (Molecular Devices, Sunnyvale, CA). ANS fluorescent spectra in the range of from 400 nm to 700 nm were collected with excitation at 365 nm.

## Additional Information

**How to cite this article**: Cheung, C. S. F. *et al*. A new approach to quantification of mAb aggregates using peptide affinity probes. *Sci. Rep.* **7**, 42497; doi: 10.1038/srep42497 (2017).

**Publisher's note:** Springer Nature remains neutral with regard to jurisdictional claims in published maps and institutional affiliations.

## Figures and Tables

**Table 1:** Peptides displayed on the selected phage clones.

Peptide clone	Peptide sequence
1	RDYHPRDHTATWGGG
2	ISSFGNPEFSTGGG
3	NYPWMAGTQSMGGG
4	GNNPLHVHHDKRGGG
5	SHTFVNEHTPPSGGG
6	WLDDQTMRNLDSGGG
7	SPLRAVAFSGAQGGG
8	GADTSKPPRFVTGGG
9	VCSPCGPVPPAKGGG
10	HMYYPGGDGRFAGGG

**Table 2:** MRM transitions used for the quantification of P02-C01.

MRM transitions (m/z)			
RDYHPRDHTATWGGG		RDYH*PRDHTATWGGG	
Precursor ion (charge)	Product ion (type, charge)	Precursor ion (charge)	Product ion (type, charge)
863.4 (+2)	786.4 (y^8^, +1)	866.4 (+2)	786.4 (y^8^, +1)
863.4 (+2)	901.4 (y^9^, +1)	866.4 (+2)	901.4 (y^9^, +1)
863.4 (+2)	940.4 (b^7^, +1)	866.4 (+2)	946.5 (b^7^, +1)
863.4 (+2)	1154.5 (y^11^, +1)	866.4 (+2)	1160.5 (y^11^, +1)
863.4 (+2)	1291.6 (y^12^, +1)	866.4 (+2)	1297.6 (y^12^, +1)
575.9 (+3)	675.8 (b^11^, +2)	577.9 (+3)	678.8 (b^11^, +2)

^*^P stands for [^13^C_5_, ^15^N_1_]-Pro amino acid residue.
